# Down-dip variations in a subducting low-velocity zone linked to episodic tremor and slip: a new constraint from ScSp waves

**DOI:** 10.1038/s41598-017-03048-6

**Published:** 2017-06-06

**Authors:** Mitsuhiro Toya, Aitaro Kato, Takuto Maeda, Kazushige Obara, Tetsuya Takeda, Koshun Yamaoka

**Affiliations:** 1 Graduate School of Environmental Studies, Nagoya University, Furo-cho, Chikusa-ku, Nagoya 464-8601 Japan; 2 Earthquake Research Institute, The University of Tokyo, 1-1-1 Yayoi, Bunkyo-ku, Tokyo 113-0032 Japan; 3 National Research Institute for Earth Science and Disaster Resilience, 3-1, Tennodai, Tsukuba-shi, Ibaraki 305-0006 Japan; 4 Hanshin Consultants Co., Ltd. 1-6-19, Imabashi, Chuo-ku, Osaka 541-0042 Japan; 5 Ministry of Education, Culture, Sports, Science and Technology, 3-2-2 Kasumigaseki, Chiyoda-ku, Tokyo 100-8959 Japan

## Abstract

Fluids are thought to play an important role in controlling episodic tremor and slow slip (ETS) in subduction zones. Therefore, constraining the along-dip distribution of fluids is necessary to better understand source mechanism of ETS, and particularly the role played by fluids in ETS generation. Here, we report clear observations of coherent *ScSp* phases with a dense seismic array in western Shikoku, Japan, where ETS has been most active over the past decade. Using numerical simulations of elastic-wave propagation to reproduce the observed *ScSp* phases, we demonstrate that, relative to shallower depths, either the V_p_/V_s_ ratio or the thickness of a low-velocity zone (LVZ) within the subducting oceanic crust increases with depth beneath the mantle wedge corner where ETS has been observed. Based on these depth dependences of the structural elements, a wide semi-ductile shear zone appears to be lubricated by high-pressurized fluid in the subducting oceanic crust at ETS source depths, and to be a key factor regulating ETS activity.

## Introduction

Among worldwide subduction zones, ETS commonly occurs on the deep extension of major tectonic boundaries that host megathrust earthquake ruptures^1, 2^. Increasing numbers of studies of seismic structure in ETS zones, including seismic tomography and receiver function analysis, indicate that high-pressurized fluids, characterized by low seismic velocities with high Poisson’s ratios, are trapped within the subducting oceanic crust^3–6^. In addition, the strong sensitivity of tremors to tidal stress changes supports the presence of high-pressurized fluids in ETS zones^7–9^. Thus, fluids trapped within subducting oceanic crust could play an important role in controlling ETS behavior^10, 11^. However, the along-dip distribution of fluids is poorly constrained, and it is unknown whether there are unique structural properties of the oceanic crust that may be related to the generation of ETS along plate boundary faults^12^.

**Figure 1:**
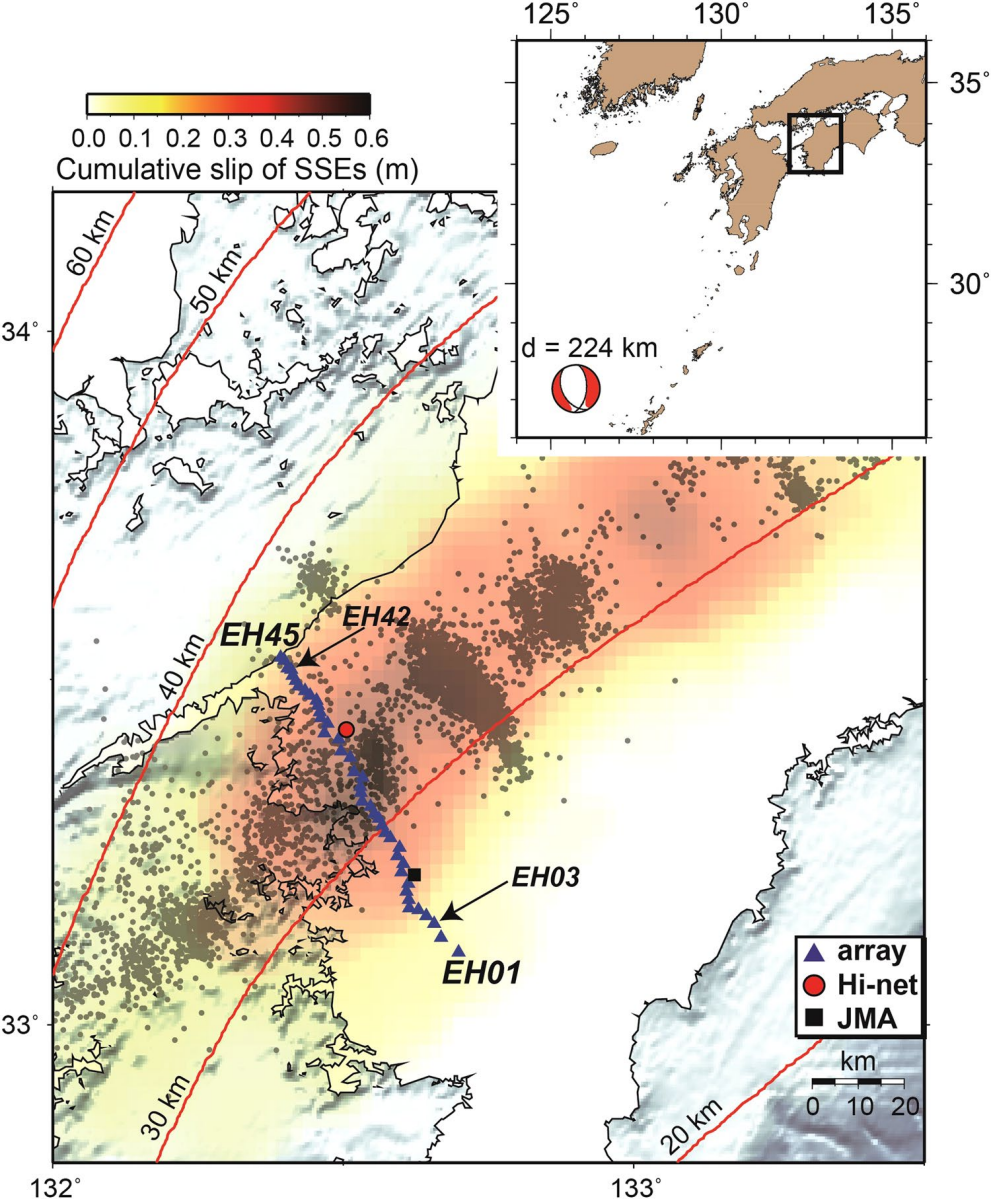
Seismotectonic setting of western Shikoku, Japan, and location of the linear seismic array used in this study. Blue symbols denote portable instruments (triangles), Hi-net station (circle), and JMA station (square). Background color scale represents the total cumulative slip of slow slip events (SSEs) between June 1996 and January 2012^14^. Gray dots are epicenters of low-frequency earthquake (LFE) determined by JMA. Red curves denote iso-depth contours of the subducting Philippine Sea Plate in the JIVSM model^20^. The inset shows the location of the study area and a large deep event analyzed in this study, along with a moment tensor (determined by F-net, NIED, Japan). Map was created using the GMT (Generic Mapping Tools, http://gmt.soest.hawaii.edu/) software package^37^.

To investigate spatial variations in structural elements within the subduction complex, we deployed a dense linear seismic array in western Shikoku, Japan, above a region extending from the locked zone to the ETS zone (Fig. 1), where ETS has been most active over the past decade^13, 14^. The array consisted of 45 portable seismic stations aligned subparallel to the direction of subduction, with a total length of ~60 km.

Here, we focus on identifying seismic phase conversions at the top of the subducting slab, for near-vertically incident *ScS* waves originating from a large, deep earthquake. The *ScSp* (*ScS*-*to*-*P* conversion) phase represents an S wave reflected at the core-mantle boundary and then converted to a *P* waves within the subduction zone. Previous studies using *ScSp* phases have investigated the geometry and physical properties of the subducting Philippine Sea (PHS) Plate^15–17^. We observed coherent *ScSp* phases on the dense seismic array, which appear to be associated with the subducting PHS Plate. To reproduce these observations, we performed numerical simulations of elastic-wave propagation using a finite difference method (FDM)^18^ that incorporated a three-dimensional structural model. The combination of coherent *ScSp* phases and numerical simulations allows us to investigate the depth dependence of Poisson’s ratios or thickness of the LVZ, and to infer the potential presence of fluids confined to the ETS zone.

## Results

**Figure 2:**
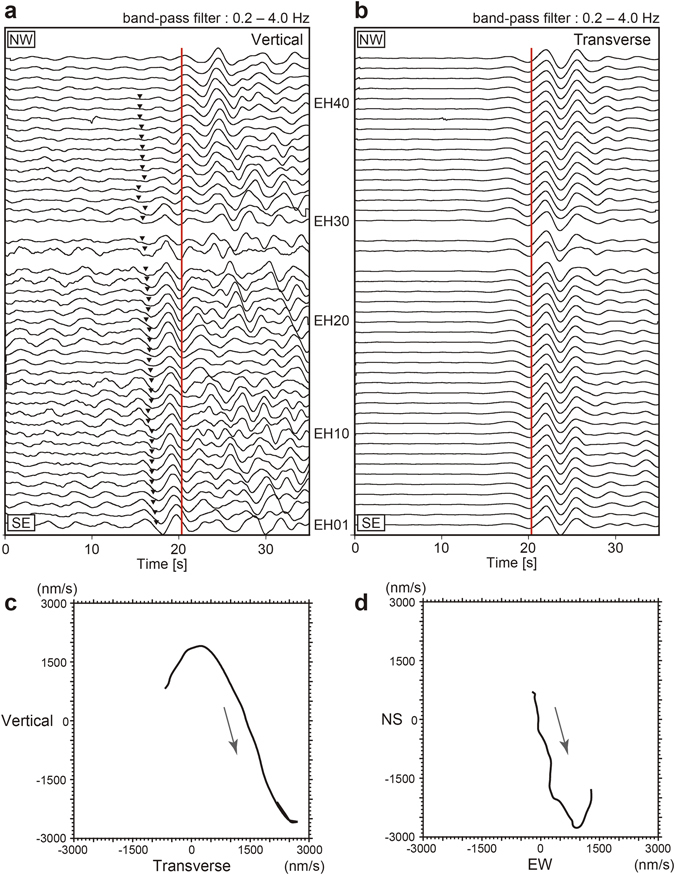
(**a)** Vertical and (**b)** transverse components of seismograms aligned around the *ScS* phase arrival. Red lines and black inverse triangles show onset times of *ScS* and *ScSp* phases, respectively. (**c)** and (**d)** show particle motions in the vertical and horizontal planes, respectively, at station EH03.

Based on comparisons of transverse and vertical component waveform data (Fig. 2a and b), we found clear, coherent signals arriving before *ScS* on the vertical components of most stations in the array. Figure 2c and d shows particle motions in the vertical and horizontal planes, respectively, for a time window that includes the *ScS* precursor observed at station EH03. Vertical motion is dominant during the precursory phase arrival. Particle motion in the horizontal plane is polarized along the orientation of the subducting plate; i.e., NW–SE. These particle motions satisfy the criteria proposed by Okada^15^ for identification of the *ScSp* phase. Therefore, these coherent precursor signals recorded by the seismic array are interpreted to be *ScSp* phases. Interestingly, travel time differences between *ScS* and *ScSp* increase from 3.0 s to 4.5 s along-dip in the direction of subduction. This means that the *ScS*-to-*ScSp* conversion point deepens to the northwest, indicating in turn that the converted waveforms propagate from the top of the subducting PHS Plate^15, 16^. The depth variations of these conversion points (28–43 km) are roughly consistent with the depth of the plate boundary interface estimated previously^19, 20^.

**Figure 3:**
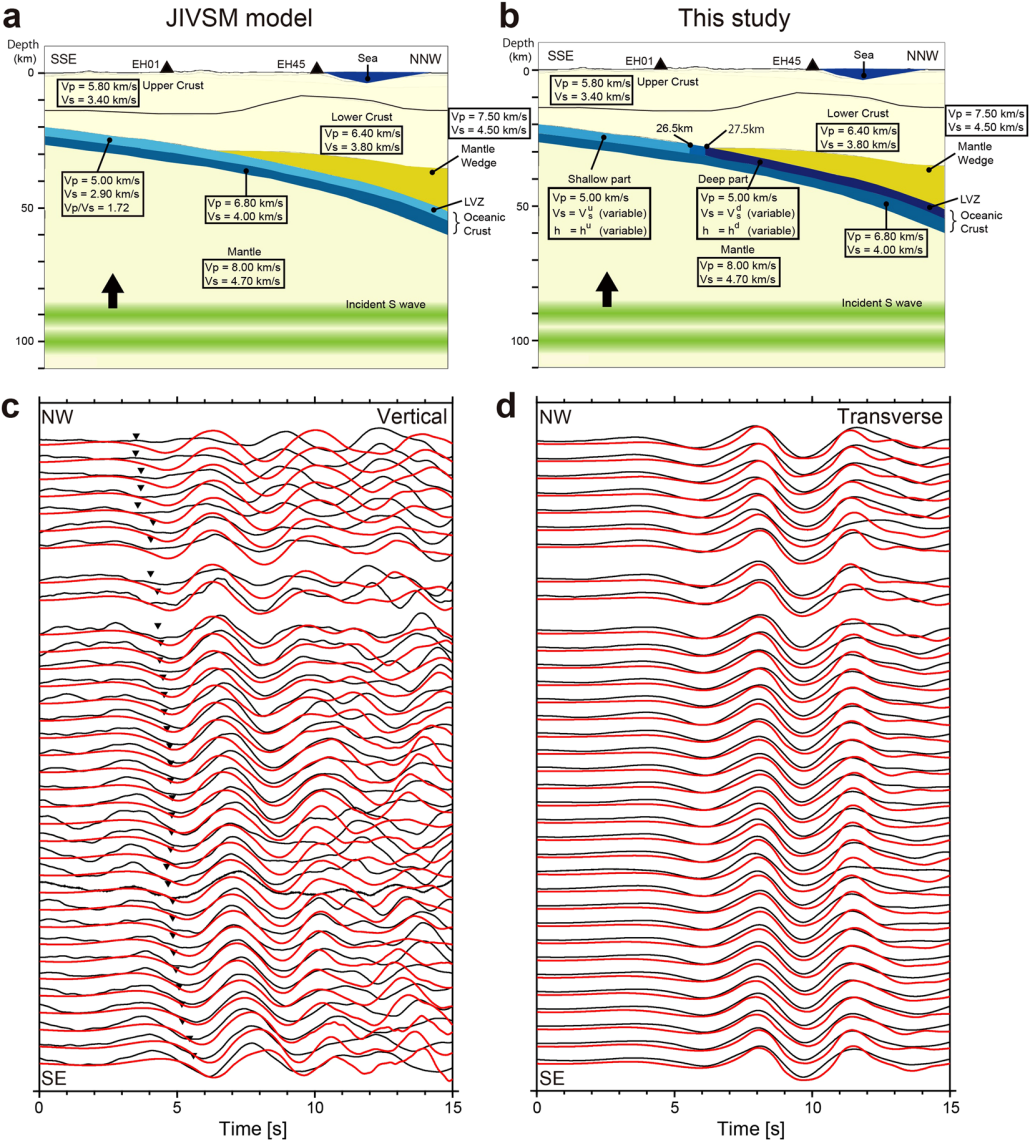
(**a)** Vertical cross-section of the initial structural model (JIVSM) along the seismic linear array, used in FDM simulations^20^. (**b)** Revised model in the present study. There are four variable parameters charactering physical properties of the LVZ. The *S*-wave velocity and the layer thickness in the partitioned LVZ are labeled V_s_^u^, *h* ^u^ in the shallower part of the LVZ, and V_s_^d^, *h* ^d^ in the deeper part, respectively. (**c)** Comparison of observed (black) and simulated (red) vertical component waveforms, from EH01 to EH35 for a constant layer thickness model. Black inverse triangles denote arrivals of observed *ScSp* phases. (**d)** Transverse components. The simulated waveforms were calculated using the best-fit parameters, V_s_^u^ = 2.9 km/s, V_s_^d^ = 2.2 km/s and *h* = 6 km. At each station, a relative amplitude between *ScSp* and *ScS* phases is saved.

We simulated the propagation of synthetic *ScSp* waveforms using the JIVSM model (Fig. 3a). The simulated *ScSp* phases were generated at multiple velocity discontinuities within the subducting oceanic crust; i.e., the top and bottom of the LVZ, and the oceanic Moho. We then aligned the simulated *ScS* phases using a procedure similar to that employed to align the observed waveform data, and compared the arrival times of simulated *ScSp* phases with our observations. However, the calculated *ScS*-*ScSp* travel time differences were systematically lower than predicted by our observations (Supplementary Figure S1). Although we replaced the plate interface model with that proposed by Hirose *et al*.^19^, the goodness of fit between the observed and calculated waveforms decreased. This is because the depth of the oceanic crust reported by Hirose *et al*.^19^ is ~3 km shallower than that of the JIVSM model, resulting in further reduction of the simulated *ScS*-*ScSp* time difference.

The predicted difference in travel times between the *ScS* and *ScSp* phases appears to be too low compared to observations. To increase the goodness of fit, we propose here that the *S*-wave velocity within the LVZ in the oceanic crust could be slower than the original values assumed in the JIVSM model, or the thickness of the LVZ could be thicker than the original JIVSM model. These features would delay only the *ScS* arrival, while keeping the *ScSp* arrival almost constant. In addition to adjusting our models, because the travel time difference between the observed and simulated waveforms was larger at the northern stations, toward which the LVZ is subducting (Figure S1), we partitioned the LVZ into shallower and deeper parts around the upper corner of the mantle wedge, taking into account the depth dependence of the physical properties. The *S*-wave velocity and the layer thickness in the partitioned LVZ are labeled V_s_^u^, *h* ^u^ in the shallower part of the LVZ, and V_s_^d^, *h* ^d^ in the deeper part, respectively (Fig. 3b).

**Figure 4:**
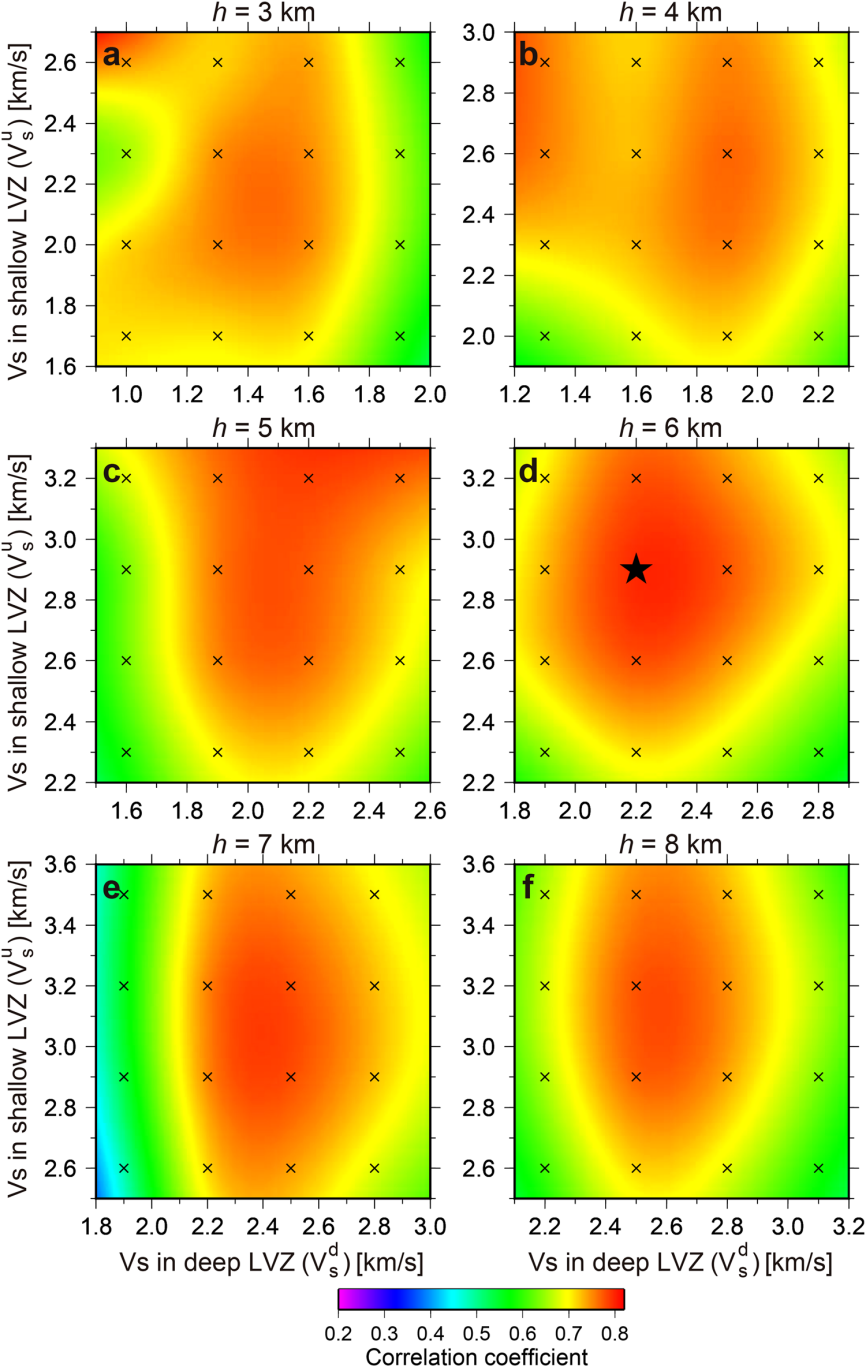
Results of a grid search with a constant layer thickness model and three free parameters: *S*-wave velocity in the shallow LVZ (V_s_^u^), *S*-wave velocity in the deep LVZ (V_s_^d^), and LVZ layer thickness (*h *=* h* ^u^ = *h* ^d^) (Figure 3b). (**a)** *h* = 3 km, (**b)** *h* = 4 km, (**c)** *h* = 5 km, (**d)** *h* = 6 km, (**e)** *h* = 7 km, (**f)** *h* = 8 km. Crosses denote calculated points, and the black star represents a peak in the cross-correlation coefficients for the best model.

First, we incorporated the depth dependence of *S*-wave velocities, assuming a constant layer thickness (*h* ^u^ = *h* ^d^ = *h*); here, we call this the constant layer thickness model. *S*-wave velocities were linearly interpolated between the shallower (V_s_^u^) and deeper (V_s_^d^) portions to ensure that no new discontinuities were introduced to the structural model (Fig. 3b). To obtain the best possible fit between simulated and observed waveforms, we conducted a grid search over the three-parameter space defined by V_s_^u^, V_s_^d^, and the layer thickness *h* of the LVZ (Fig. 4). To quantify the fit, we averaged the cross-correlation coefficients between observed and simulated *ScSp* phases centered in a 7 s time window over all stations with identifiable *ScSp* phase onsets (EH01–EH35), since *ScSp* phases at the northern stations are emergent. We then used a contour plot to search for the best-fit parameters.

**Figure 5:**
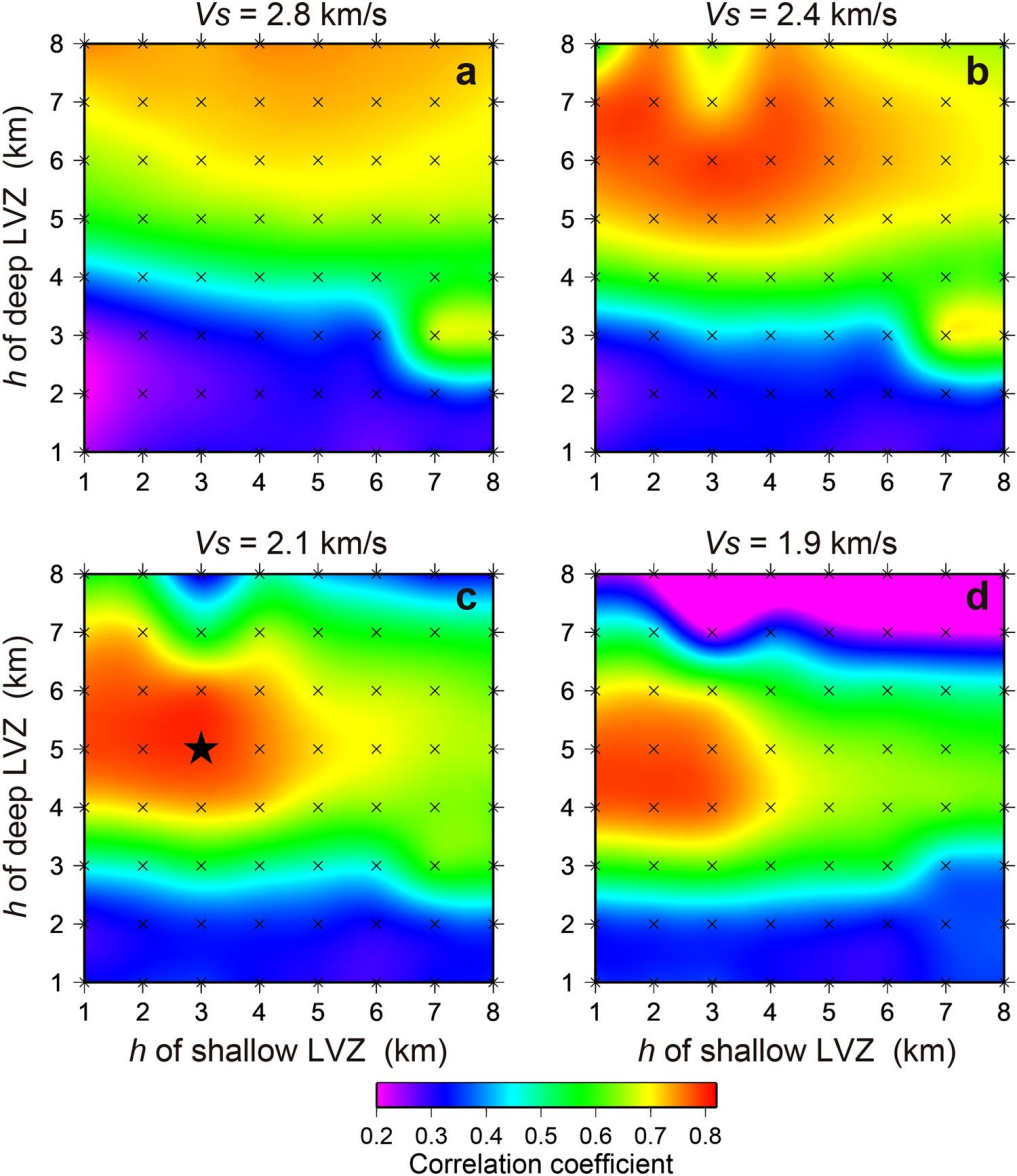
Results of a grid search with an assumed constant velocity and three free parameters: thicknesses of the shallow and deep LVZs (*h* ^u^, *h* ^d^), and *S*-wave velocity (V_s_ = V_s_^u^ = V_s_^d^ ) (Fig. 3b). (**a)** V_s_ = 2.8 km/s, (**b)** V_s_ = 2.4 km/s, (**c)** V_s_ = 2.1 km/s, (**d)** V_s_ = 1.9 km/s. Crosses denote calculated points, and the black star represents a peak in the cross-correlation coefficients for the best model.

As an alternative model, we assumed a depth-dependent LVZ thickness around the upper corner of the mantle wedge with constant seismic velocities (i.e., V_s_^u^ = V_s_^d^ = V_s_ with a constant V_p_/V_s_ ratio within the LVZ.) Hereafter, we call this the constant velocity model. To obtain the best possible fit between simulated and observed waveforms, we conducted another set of grid searches over the three-parameter space defined by *h* ^u^, *h* ^d^, and V_s_ within the LVZ (Fig. 5).

From these grid search results, within reasonable ranges of the parameters (Figs 4 and 5), the best-fit model has V_s_^u^ = 2.9 km/s, V_s_^d^ = 2.2 km/s, and *h* = 6 km for the constant layer thickness model, or, *h* ^u^ = 3 km, *h* ^d^ = 5 km, and V_s_ = 2.1 km/s for the constant velocity model. The averaged cross-correlation coefficients reached 0.8 for each best-fit model. Given the trade-off between the depth dependence of *S*-wave velocities and LVZ thickness, the difference in arrival times of the *ScSp* and *ScS* waves means that each best-fit model is able to adequately explain the observed waveforms. Figure 3c and d compare the observed and simulated *ScSp* and *ScS* waves, calculated using the best-fit parameters for the constant layer thickness model (black star in Fig. 4d). Along the seismic array, from south (EH01) to north (EH35), the simulated *ScSp* arrivals correlate well with observed waveforms (Fig. 3c). In summary, these modeling results suggest that either the S-wave velocities within the LVZ decrease or the thickness of the LVZ increases at depths greater than around the continental Moho; i.e., at ETS source depths.

## Discussion

Based on comparison of transverse and vertical waveform data (Fig. 2a and b), we identified clear, coherent *ScSp* phase arrivals along a seismic array in western Shikoku, Japan, which converted near the PHS Plate boundary. Although some previous studies have detected *ScSp* phases associated with the PHS Plate^16, 17^, it was difficult to precisely illustrate the depth variations of the plate boundary, due to the sparseness of the seismic network at the time of these previous works. The present study is the first to obtain a spatially continuous image of the PHS Plate boundary using *ScSp* phases with fine spatial resolution.

We performed numerical simulations of elastic wave propagation to reproduce observed *ScSp* phases, using the JIVSM velocity model as an initial (reference) model. However, the calculated *ScS*-*ScSp* travel time differences were systematically smaller than those predicted by our observations. To improve the goodness of fit, we conducted an extensive parameter search of shear wave speeds (V_s_^u^, V_s_^d^) and LVZ thicknesses (*h* ^u^, *h* ^d^) within the oceanic crust (Figs 4 and 5). However, it could be claimed that the interpretations are nonunique, because spatial variations of the other parameters in the velocity model, especially within the overriding plate, can also explain the features of the observed *ScSp* phases. To address this possibility, we conducted additional numerical simulations while changing other model parameters with the physical properties of the LVZ held constant.

As a first case, we considered serpentinization of the mantle wedge above the ETS source. We simulated the *ScSp* phases by changing both V_p_ and V_s_ in the mantle wedge while assuming several degrees of serpentinization, from dunite to antigorite serpentinite (0%, 50%, and 100% serpentinization)^21^, as well as from dunite to lizardite serpentinite (30%, 60%, and 100% serpentinization)^21^. However, the simulated waveforms did not fit the observed waveforms well for the two species of serpentinite (Supplementary Figures S2 and S3), because serpentinization in the mantle wedge corner reduces the *ScS*-*ScSp* travel time difference along the linear seismic array. In addition, there is no significant variation of crustal seismic structure in the overlying plate in the studied area, based on regional tomographic images^22^ revealed by a dense seismic network in Japan (Hi-net). Therefore, heterogeneous structure in the mantle wedge or in the crust of the overlying plate is not sufficient to explain the observed features of *ScS*-*ScSp* travel time difference along the dense seismic array.

In the present analysis, we assumed a constant P-wave velocity of 5 km/s in the LVZ for all waveform simulations. To investigate the sensitivity of the results to the assumed V_p_ value, we conducted a grid search over a V_p_ range of 4–6 km/s in the LVZ, following the procedure described above. For the constant layer thickness model (*h* = 6 km), the S-wave velocities of the LVZ at greater depths are lower than those at shallower depths (Supplementary Figure S4), regardless of the assumed V_p_ value. Therefore, we consider the present conclusions to be robust with respect to the assumed V_p_ within the LVZ. Presumably this robustness arises because we focus on the *ScS*-*ScSp* travel time difference, which has only a weak dependence on the absolute value of the velocity.

**Figure 6:**
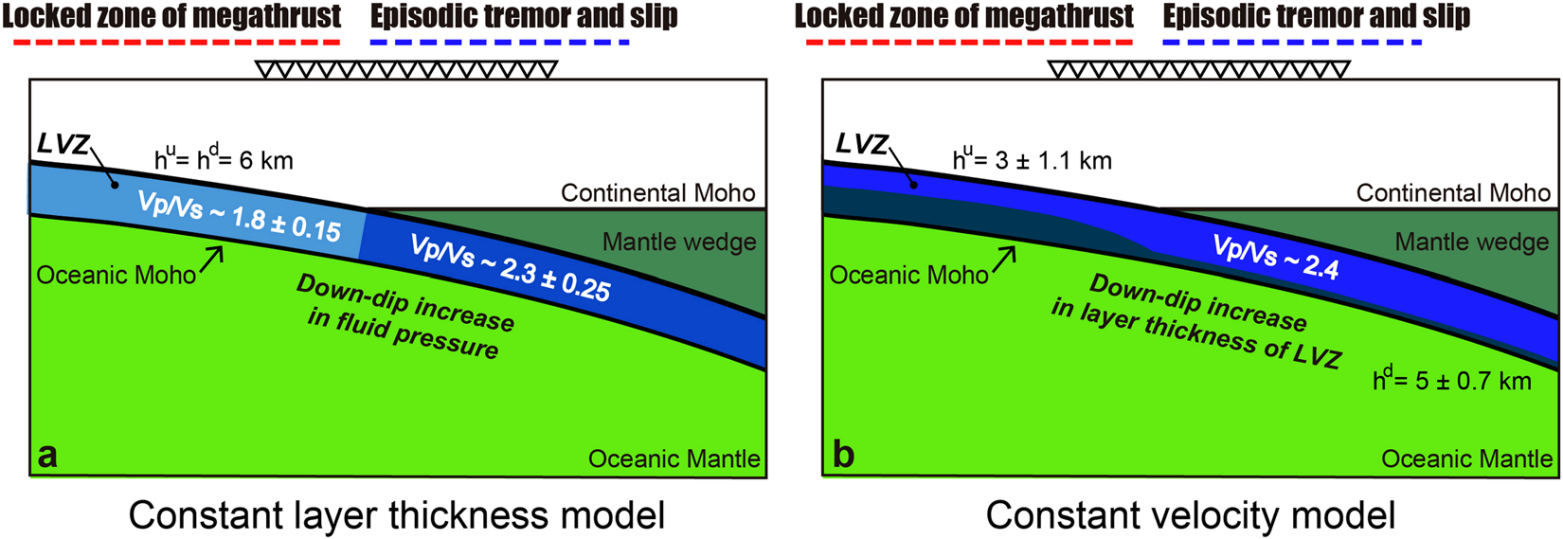
Schematic figures showing depth variations of structural elements in the LVZ, relative to the locations of ETS. (**a)** Fluid pressure changes in the LVZ inferred from the constant layer thickness model. (**b)** Layer thickness changes in the LVZ derived from the constant velocity model. To evaluate uncertainties for the model parameters, we calculated the standard deviation of the parameters with the cross-correlation coefficient greater than 0.72 for each best-fit model (Figs 4 and 5).

From an extensive parameter search of shear wave speeds (V_s_^u^, V_s_^d^) and LVZ thicknesses (*h* ^u^ = *h* ^d^ = *h*) within the oceanic crust (Fig. 4), the best-fit parameters for a constant layer thickness model were estimated to be V_s_^u^ = 2.9 km/s, V_s_^d^ = 2.2 km/s, and *h* ^u^ = *h* ^d^ = 6 km. The resulting V_p_/V_s_ ratio has a high value (~2.3) at depths of >~30 km. Interestingly, at depths greater than the mantle wedge, the shear wave velocity within the LVZ is slower than that of the shallower portion of the LVZ for any reasonable LVZ thickness (Fig. 4). This result indicates that the reduction in shear wave speeds at greater depths is a plausible and robust feature within a reasonable range of layer thicknesses. The reduction in shear wave velocity leads to an increase in V_p_/V_s_ ratio (Poisson’s ratio) within the LVZ beneath the mantle wedge corner where ETS has been observed (Fig. 6a), because we assume V_p_ is constant within the LVZ. The incremental change in the V_p_/V_s_ ratio in the deep portion of the LVZ is around 0.5 with the parameters that yield the best-fit constant layer thickness model.

With a constant LVZ thickness, the active ETS zone roughly overlaps the high-V_p_/V_s_ layer within the oceanic crust beneath the mantle wedge corner (Fig. 6a). This feature matches well with previous studies of seismic velocity models in SW Japan, which have shown that ETS takes place above low-velocity layers of oceanic crust with high V_p_/V_s_ ratios, as well as beneath the mantle wedge corner^3, 5, 23^. Because this V_p_/V_s_ ratio is significantly higher than expected for a fully hydrated (about 5 wt%) MORB^24^, the high-V_p_/V_s_ zone implies the presence of high-pressurized fluids released by progressive metamorphic dehydration reactions in the oceanic crust around the ETS zone^3–6^.

Alternatively, the best-fit model with a constant *S*-wave velocity has *h* ^u^ = 3 km, *h* ^d^ = 5 km, and V_s_^u^ = V_s_^d^ = 2.1 km/s; i.e., V_p_/V_s_ ratio = 2.4 (Fig. 6b). The V_p_/V_s_ ratio is higher than that derived from the constant layer thickness model. This also indicates that fluids released by dehydration reactions could be trapped in the LVZ from shallow to deep depths. Regardless of the absolute value of the assumed LVZ *S*-wave velocity, the layer thickness increases below the depth of the mantle wedge corner (Fig. 5). The incremental change in LVZ thickness is 2–3 km for the good-fit models, which is almost double the thickness of the shallow LVZ. A similar increase in layer thickness of the LVZ near ETS source depths has been suggested to explain the results of wide-angle seismic reflection surveys in the Nankai subduction zone^25, 26^. In addition to Nankai, in the Cascadia, Chile, Alaska, and Mexico subduction zones^27–31^ there seems to be a positive correlation between the thickness of the reflection band near the plate boundary and the degree of unlocking between the overlying and subducting plates. The locked region along the plate boundary fault shows a thin, sharp reflection, whereas the deeper part, characterized by aseismic slip, exhibits a thick reflection band^26, 29, 30^. Based on geological studies of plate boundary faults in exhumed subduction-related rock assemblages^32^, the increased thickness of the LVZ near ETS source depths is attributed to a well-developed ductile shear zone, consisting mainly of trench-fill sediments. The thicker shear zone may accommodate shear strain by distributed flow deformation, lubricated by fluids trapped in the LVZ^32, 33^.

Although it is difficult to distinguish between these different models, the structures within the oceanic crust at ETS source depths are commonly characterized by a relatively thick layer (4~6 km) with high V_p_/V_s_ ratio (>~2.3) (Fig. 6). This feature indicates that a wide semi-ductile shear zone is lubricated by high-pressurized fluid ^31^in the subducting oceanic crust at ETS source depths. These down-dip variations in physical properties might play an important role in regulating ETS activity.

## Methods

We deployed a dense linear seismic array from October 2011 to April 2013 on western Shikoku Island, SW Japan (Fig. 1). The array consisted of 45 three-component seismometers recording in continuous acquisition mode: 43 instruments with a natural frequency of 1 Hz, and 2 with a natural frequency of 0.2 Hz. We also used permanent stations near the array from the Hi-net network, operated by the National Research Institute for Earth Science and Disaster Prevention (NIED; Okada *et al*.^34^, and stations of the Japan Meteorological Agency (JMA). During the deployment period, we visually inspected seismograms of Mw ≥ 6 deep earthquakes with focal depths greater than 90 km and epicentral distances Δ < 25°; within this range, no other phases are expected to arrive that could be misidentified as *ScSp* ^17^. Using this procedure, we found a clear *ScS* precursor following an M_w_ 6.9 earthquake that occurred on 8 November 2011 in the backarc of the Ryukyu Trench, at a focal depth of 224.8 km (Fig. 1). We corrected the frequency response of the seismometers using a recursive time domain filter^35^, then applied a 0.2–4.0 Hz band-pass filter to the corrected seismograms. Using the transverse components of rotated seismograms from the array, we shifted the *ScS* phases relative to the arrival at station EH42 (Fig. 1) by cross-correlating the *ScS* phase from EH42 with other *ScS* waveforms to achieve the maximum correlations. In other words, we aligned the seismograms as if the *ScS* phase arrived simultaneously. The vertical component data at each station were then time shifted by the corresponding time lags, relative to EH42 (Figure 2a).

We performed numerical simulations of elastic-wave propagation to reproduce the observed *ScSp* phases using the three-dimensional FDM code of Maeda *et al*.^18^. Figure 3a shows a vertical cross-section of the Japan Integrated Velocity Structure Model (JIVSM^20^) used in the numerical simulations, where P- and *S*-wave velocities (V_p_, V_s_) in each layer are labeled. This model was constructed by integrating seismic structures deduced from extensive refraction/reflection experiments, gravity surveys, surface geology, borehole logging data, microtremor surveys, and earthquake ground motion records. The thickness of the LVZ within the subducting oceanic crust was assumed to be 3 km in the JIVSM model. The target area comprised a zone 150 × 150 km in horizontal extent, and 85 km in depth, which was discretized in grid intervals of 0.1 km in each direction. As an incident wave, we used an *SH* plane wave with 20 km wavelength, propagating vertically toward the seismic array, mimicking the *ScS* wave, whose incidence angle is nearly vertical^36^. The polarization azimuth of the synthetic *SH*-wave was set to N29°W, which roughly corresponds to the polarizations of the *ScS* phases observed in this study (Fig. 2d).

## Electronic supplementary material

Supplementary Files
