# Supplementary material for: Down-dip variations in a subducting low-velocity zone linked to episodic tremor and slip: a new constraint from ScSp waves
Source: Sci Rep. 2017 Jun 6;7:2868. doi: 10.1038/s41598-017-03048-6 (PMC5460158; doi:10.1038/s41598-017-03048-6)
Supplement: Supplementary file 1 — Supplementary Files [file 41598_2017_3048_MOESM1_ESM.pdf]

Supplementary Information

Down-dip variations in a subducting low-velocity zone linked to episodic tremor and slip: a new constraint from ScSp waves

Mitsuhiro Toya[1,4], Aitaro Kato[1, 2*], Takuto Maeda[2], Kazushige Obara[2], Tetsuya Takeda[3,5] and Koshun Yamaoka[1]

1: Graduate School of Environmental Studies, Nagoya University, Nagoya, Japan.

2: Earthquake Research Institute, The University of Tokyo, Tokyo, Japan.

3: National Research Institute for Earth Science and Disaster Resilience, Tsukuba, Japan.

4: Present address: Hanshin Consultants Co., Ltd. Osaka, Japan.

5: Present address: Ministry of Education, Culture, Sports, Science and Technology, Tokyo, Japan.

*corresponding. akato@eri.u-tokyo.ac.jp

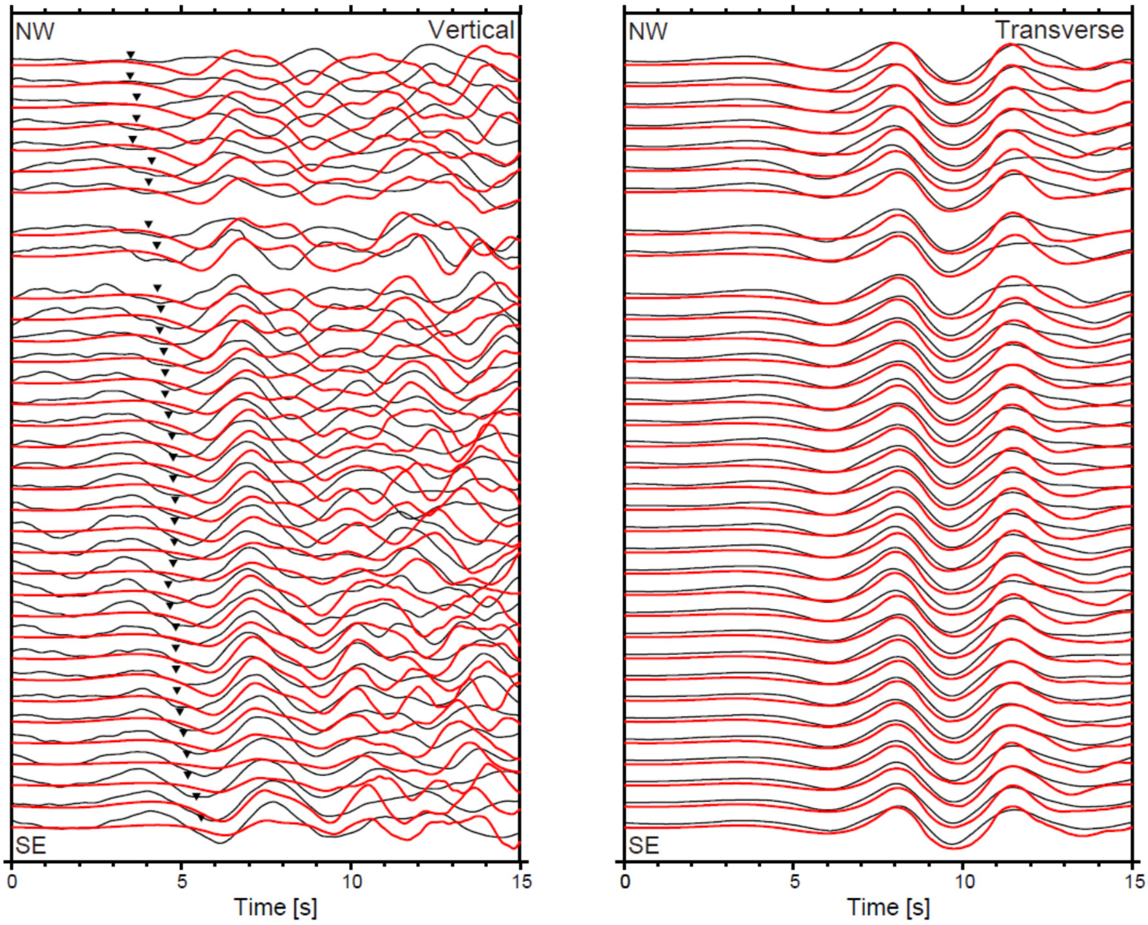

Supplementary Figure S1. Comparison of observed (black) and simulated (red) vertical and transverse component waveforms, from EH01 to EH35, using the JIVSM model[1]. Black inverse triangles denote arrivals of observed *ScSp* phases. The calculated *ScS*-*ScSp* travel time differences is systematically lower than predicted by our observations. The averaged cross-correlation coefficient between the observed and simulated waveforms is 0.34.

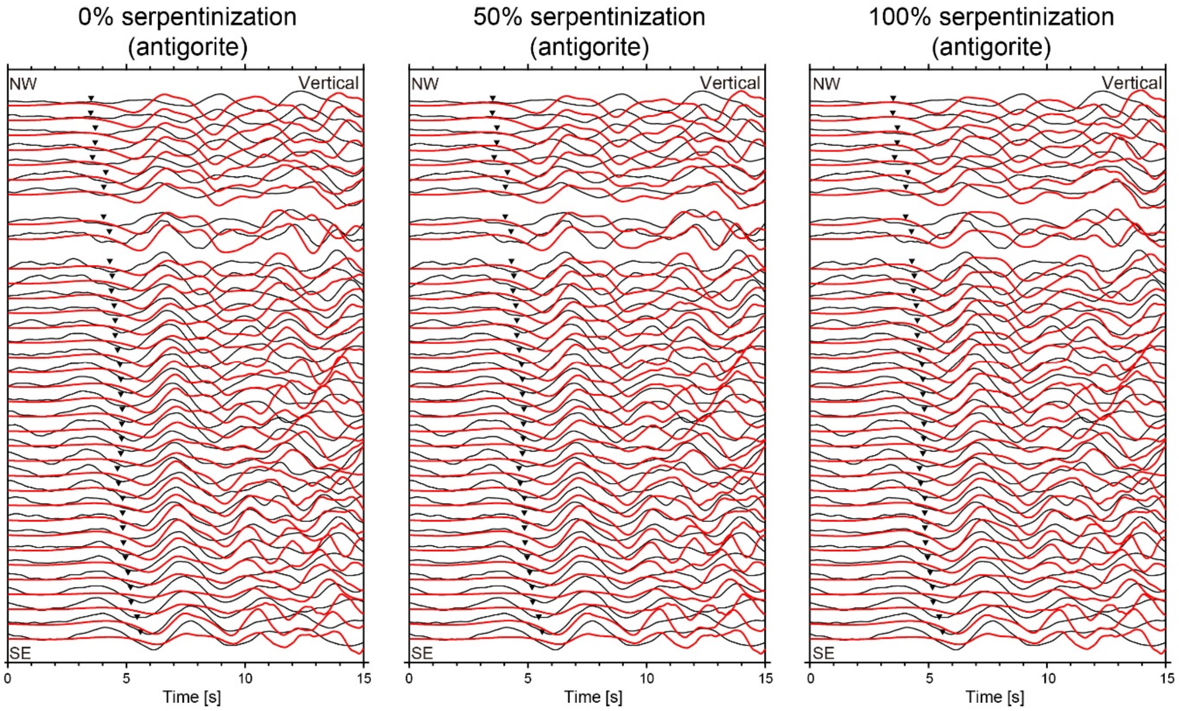

Supplementary Figure S2. Comparison of observed (black) and simulated (red) vertical component waveforms, from EH01 to EH35, assuming several degrees of serpentinization, from dunite to antigorite serpentinite[2] (0 %, 50 %, and 100 % serpentinization). The assumed V_p/V_s ratio for each serpentinization is 1.76, 1.78 and 1.83. Black inverse triangles denote arrivals of observed *ScSp* phases. The simulated waveforms did not fit the observed waveforms well.

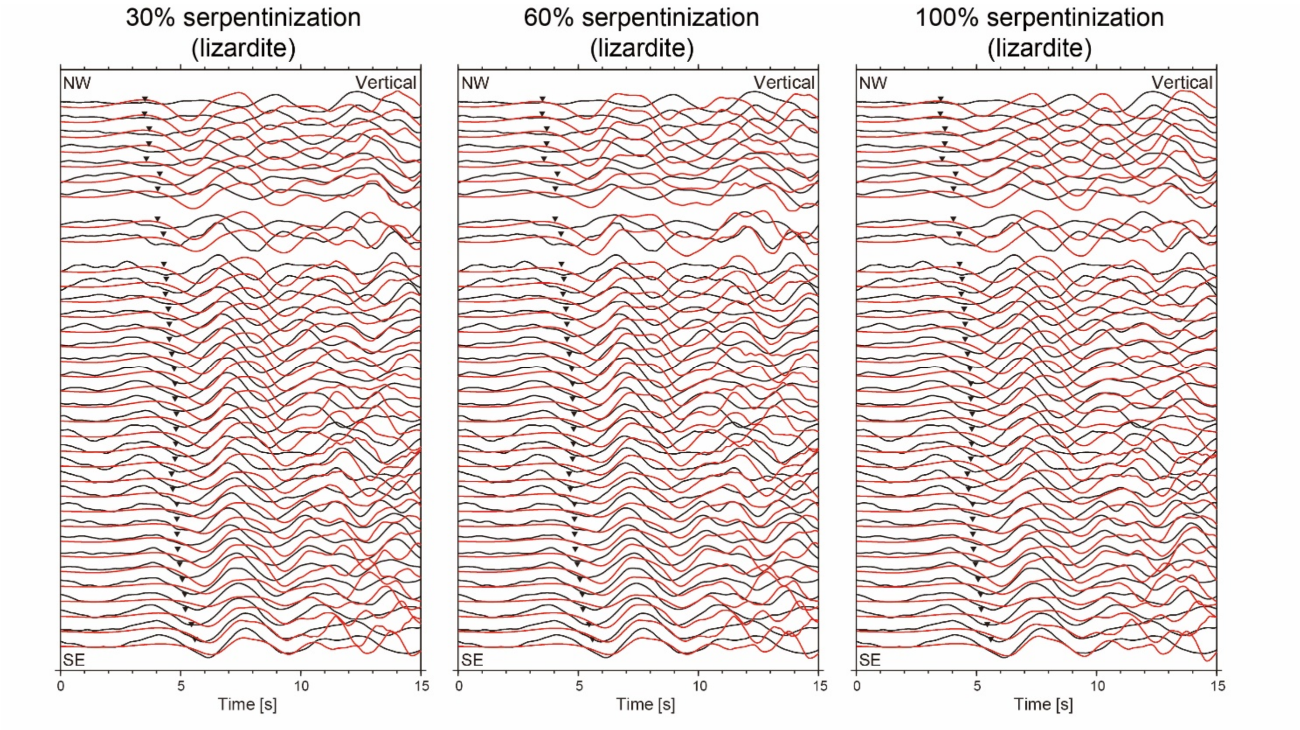

Supplementary Figure S3. Comparison of observed (black) and simulated (red) vertical component waveforms, from EH01 to EH35, assuming several degrees of serpentinization, from dunite to lizardite serpentinite[2] (30 %, 60 %, and 100 % serpentinization). The assumed V_p/V_s ratio for each serpentinization is 1.84, 2.07 and 2.17. Black inverse triangles denote arrivals of observed *ScSp* phases. The simulated waveforms did not fit the observed waveforms well.

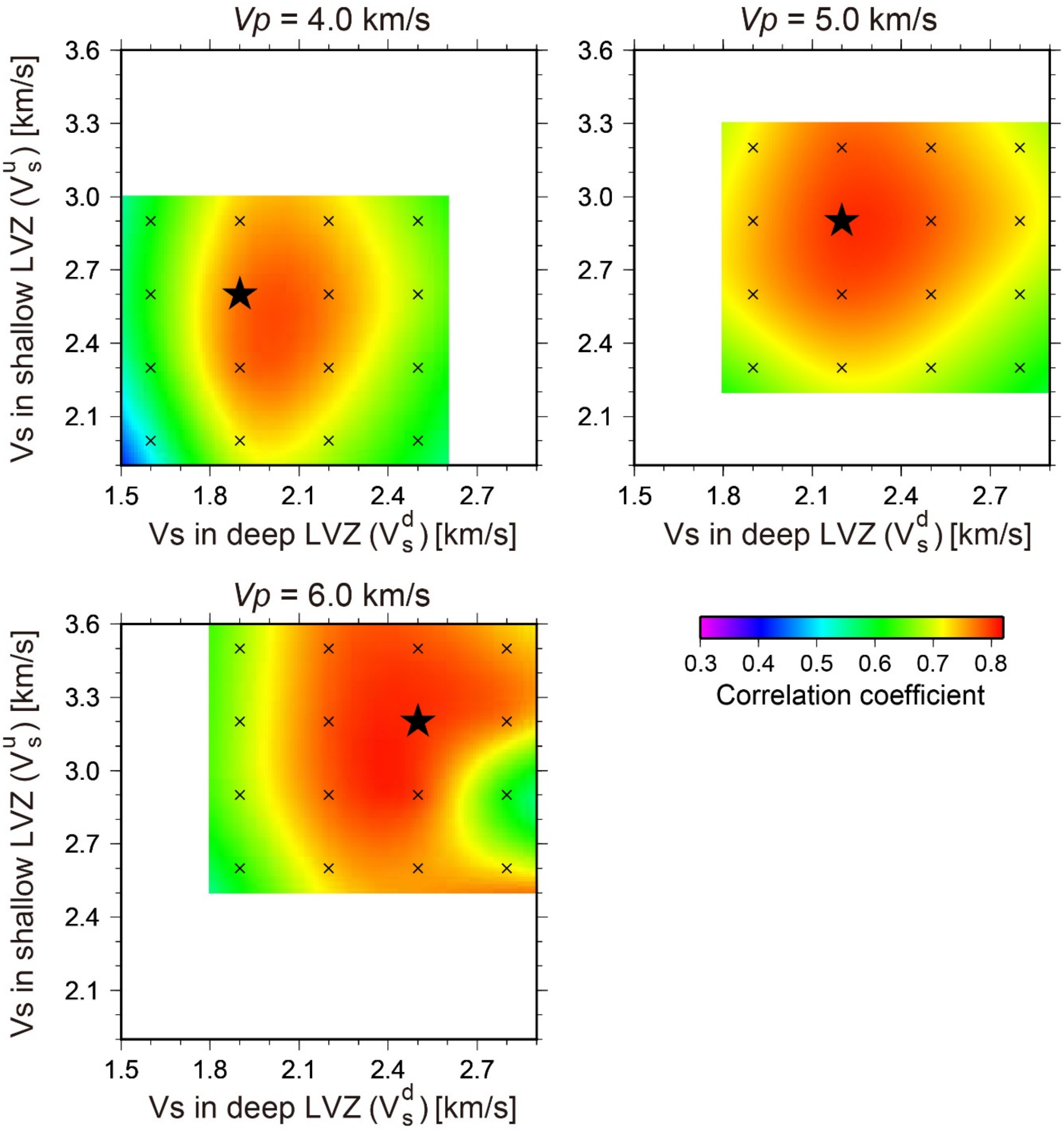

Supplementary Figure S4. Results of a grid search with a constant layer thickness model (h = 6 km) for different assumed P-wave velocities and two free parameters: S-wave velocity in the shallow LVZ (V_s^u) and S-wave velocity in the deep LVZ (V_s^d). Crosses denote calculated points, and the black star represents a peak in the cross-correlation coefficients for the best model for each assumed value of the P-wave velocity. The S-wave velocities of the LVZ at greater depths are lower than those at shallower depths, regardless of the assumed V_p value. Therefore, we consider the present conclusions to be robust with respect to the assumed V_p within the LVZ.

Supplementary references

1. Koketsu, K., Miyake, H. & Suzuki, H. Japan Integrated Velocity Structure Model version 1, *Proceedings of the 15th World Conference on Earthquake Engineering*, Paper No.1773 (2012).

2. Christensen, N. Serpentinites, peridotites, and seismology. *Int. Geol. Rev.* **46**(9), 795–816, doi:10.2747/0020-6814.46.9.795 (2004).
